# Reduced host-specificity in a parasite infecting non-littoral Lake Tanganyika cichlids evidenced by intraspecific morphological and genetic diversity

**DOI:** 10.1038/srep39605

**Published:** 2016-12-22

**Authors:** Nikol Kmentová, Milan Gelnar, Monika Mendlová, Maarten Van Steenberge, Stephan Koblmüller, Maarten P. M. Vanhove

**Affiliations:** 1 Department of Botany and Zoology, Faculty of Science, Masaryk University, Kotlářská 2, 611 37 Brno, Czech Republic; 2 Biology Department, Royal Museum for Central Africa, Leuvensesteenweg 13, B-3080 Tervuren, Belgium; 3 Institute of Zoology, University of Graz, Universitätsplatz 2, A-8010 Graz, Austria; 4 Laboratory of Biodiversity and Evolutionary Genomics, Department of Biology, University of Leuven, Ch. Deberiotstraat 32, B-3000 Leuven, Belgium; 5 Institute of Vertebrate Biology, Academy of Sciences of the Czech Republic, Květná 8, 603 65 Brno, Czech Republic; 6 Hasselt University, Centre for Environmental Sciences, Research Group Zoology: Biodiversity & Toxicology, Agoralaan Gebouw D, B-3590 Diepenbeek, Belgium

## Abstract

Lake Tanganyika is well-known for its high species-richness and rapid radiation processes. Its assemblage of cichlid fishes recently gained momentum as a framework to study parasite ecology and evolution. It offers a rare chance to investigate the influence of a deepwater lifestyle in a freshwater fish-parasite system. Our study represents the first investigation of parasite intraspecific genetic structure related to host specificity in the lake. It focused on the monogenean flatworm *Cichlidogyrus casuarinus* infecting deepwater cichlids belonging to *Bathybates* and *Hemibates*. Morphological examination of *C. casuarinus* had previously suggested a broad host range, while the lake’s other *Cichlidogyrus* species are usually host specific. However, ongoing speciation or cryptic diversity could not be excluded. To distinguish between these hypotheses, we analysed intraspecific diversity of *C. casuarinus*. Monogeneans from nearly all representatives of the host genera were examined using morphometrics, geomorphometrics and genetics. We confirmed the low host-specificity of *C. casuarinus* based on morphology and nuclear DNA. Yet, intraspecific variation of sclerotized structures was observed. Nevertheless, the highly variable mitochondrial DNA indicated recent population expansion, but no ongoing parasite speciation, confirming, for the first time in freshwater, reduced parasite host specificity in the deepwater realm, probably an adaptation to low host availability.

Host specificity is one of the basic biological factors influencing the life cycle and diversity of parasitic organisms^1^. It is highly variable among groups and within taxa, ranging from strict specialist to generalist species, but always limited by the occurrence of potential hosts^2^. Host specificity is characterised by a trade-off of costs and benefits. While specialists have evolved specific adaptations to their host and therefore maximise profits, generalist species infecting a broad range of hosts are less affected by possible host extinction.

But to what extent is this important aspect of parasite biodiversity dependent on host ecology? The capability of a parasite to infect a host is determined by their co-evolutionary history and also ecological determinants such as host species longevity, stability and seasonality of a particular ecosystem. However, it seems that host species’ ecological similarity is more important than host phylogeny^3^.

Lower host-specificity affected by decreasing host population density in deepwater habitats has been documented in marine environments^4^,^5^,^6^ but never in freshwater systems. Decreased host-specificity in marine pelagic deepwater habitats was proposed to increase the chance of finding a host if host species exhibit low population densities, which is characteristic for most deepwater taxa^4^,^5^,^6^. In the present study we focus on yet unexplored parasite host choice patterns in the non-littoral habitat (i.e. the pelagic and deepwater zone) of one of the biggest and in terms of biodiversity most exceptional freshwater ecosystems in the world.

Lake Tanganyika, situated in the East African Rift Valley, is the second deepest and second oldest lake in the world. It is known for its remarkable species diversity characterised by rapid radiation processes in many vertebrate and invertebrate taxa^7^, including parasitic flatworms that infect cichlids^8^. Therefore, it has been intensively studied for many decades. Although the first record of parasitic flatworms in Lake Tanganyika stems from a study on cestodes from 1914^9^, the knowledge about the diversity and role of parasitic organisms in this unique environment is still poor and fragmentary. In the last years, parasitological research in the lake has mainly focused on the monogenean fauna of its cichlids and the number of described species is increasing^10^,^11^,^12^,^13^,^14^,^15^,^16^,^17^,^18^. Monogenea van Beneden, 1858 is one of the most species-rich groups of Platyhelminthes^19^,^20^ with more than 3,500 already described species^21^. Most monogeneans are ectoparasites that infect the body or gills of freshwater and marine fishes. One species has a mammalian host and some have also colonised invertebrates or adopted an endoparasitic lifestyle inside fishes, turtles or amphibians^22^.

The most important attachment organ of monogeneans is the opisthaptor, which is located posteriorly and which contains sclerotized structures such as hooks, clamps or suckers^23^. The evolutionary expansion of this parasitic group is related to the opisthaptor diversity and its adaptability to different hosts and infection sites^24^. Whereas the haptoral region is characteristic of species groups or lineages, the morphology of the male copulatory organ (MCO) is important for species-level diagnosis in many groups of Monogenea^22^. The ecological, behavioural and phylogenetic diversity of cichlid fishes, especially in Lake Tanganyika^25^,^26^, make them ideal models for investigating parasite speciation mechanisms such as the influence of host ecology on parasite diversity^27^,^28^,^29^. Cichlids (Teleostei, Cichlidae) form one of the most diverse vertebrate families with around 2,200 known species^30^. In each of the African Great Lakes, hundreds of endemic species evolved within a short period of time^31^,^32^,^33^,^34^,^35^. Currently, 13 monogenean genera are known to infect cichlid species and six of these have been observed on African representatives^36^. *Cichlidogyrus* (Monopisthocotylea, Dactylogyridae) is the most species rich, with 102 representatives recorded from 88 different host species^10^,^11^,^12^,^13^,^14^,^15^,^18^,^37^,^38^,^39^,^40^,^41^,^42^. This genus displays variation in host-specificity and contains generalist but also strictly specialist species^39^,^43^. In Lake Tanganyika, most species of *Cichlidogyrus* described to date are strict or intermediate specialists^8^,^10^,^12^,^13^,^15^ following the terminology used in Mendlová & Šimková^43^. While strict specialists infect only a single host species, intermediate specialists parasitise on two or more congeneric host species and intermediate generalist infect heterogeneric host species from the same tribe. The host range of generalists includes two or more hosts from different tribes. A complete list of *Cichlidogyrus* species from Lake Tanganyika with their host species is provided in Table 1. It was suggested that the relatively high degree of monogenean host-specificity is the result of adaptive processes related to their direct life cycle and to the tight co-evolutionary interactions with their hosts^24^,^44^,^45^, depending on the species-specific response to both mechanical structures and the chemical composition of fish tissue^46^,^47^. To date, 24 species of *Cichlidogyrus* have been described from 20 different cichlid host species from Lake Tanganyika^10^,^11^,^12^,^13^,^14^,^15^,^18^. Only three of these have been reported from the benthopelagic and truly pelagic deepwater environment: *Cichlidogyrus brunnensis, C. attenboroughi* Kmentová, Gelnar, Koblmüller & Vanhove, 2016 and *C. casuarinus* Pariselle, Muterezi Bukinga & Vanhove, 2015. The present study focuses on exploring the intraspecific diversity of *C. casuarinus* infecting two deepwater cichlid genera, *Bathybates* Boulenger, 1898 and *Hemibates* Regan, 1920. These genera constitute the endemic tribe Bathybatini Poll, 1986. Until recently, also the genus *Trematocara* was included in the tribe^48^, but genome-wide data^49^ suggest that Poll’s^52^ original classification into Bathybatini, comprising the genera *Bathybates* and *Hemibates*, and Trematocarini, consisting of *Trematocara*, is more reasonable than alternative classifications^34^,^48^,^51^,^53^. The tribe Bathybatini contains eight currently recognised benthopelagic and truly pelagic species in two deeply divergent genera^48^,^49^,^53^ (see Fig. 1). Whereas *Bathybates* species are chiefly piscivorous, *Hemibates* has a broader diet that also includes shrimps. With the exceptions of *B. ferox*, which has not been recorded below 70 meters and *B. horni*, of which no information is available, all species within these two genera have maximal recorded depth ranges ranging from 160 down to 210 meters. Hence, some of these species occur just above the lake’s anoxic zone^54^. Morphological and genetic data were collected to test the hypothesis of Pariselle *et al*.^15^, who suggested that *Cichlidogyrus casuarinus* has a broader host range than its congeners in Lake Tanganyika because it infects pelagic deepwater hosts. Here, the *Cichlidogyrus* host-specificity in the deepwater habitat in Lake Tanganyika is tested for all fish hosts within the presumed host range of *C. casuarinus*, potentially the first intermediate generalist of *Cichlidogyrus* reported for Lake Tanganyika (see Table 1), on a lake-wide geographical scale. However, in other monogeneans there are reports of cryptic speciation, with allegedly generalist monogeneans representing a complex of more host-specific cryptic species^28^ or incipient speciation, with haplotypes or morphotypes of the same generalist species preferring a certain host species^55^. These scenarios can only be verified by studying *C. casuarinus* at the intraspecific level. There are only few studies about African monogeneans focusing on intraspecific aspects^56^. Here, the *Cichlidogyrus* host-specificity in the non-littoral habitat in Lake Tanganyika is tested for all fish species within the presumed host genera of *C. casuarinus* and on a lake-wide geographical scale. Multivariate statistic approaches of morphological characters and genetic characterisation using markers with different rates of molecular evolution were used to answer the following questions:

1. How broad is the host range of this parasite species among members of the Bathybatini?
2. Is there any morphological intraspecific variation?
3. Does the apparently broad host range of *Cichlidogyrus casuarinus* infecting *Bathybates* and *Hemibates* reflect cryptic speciation or a lack of host preference?
4. What is the population structure and recent demographic history of this deepwater species of *Cichlidogyrus*?

## Results

### Morphological species identification

**Figure 1:**
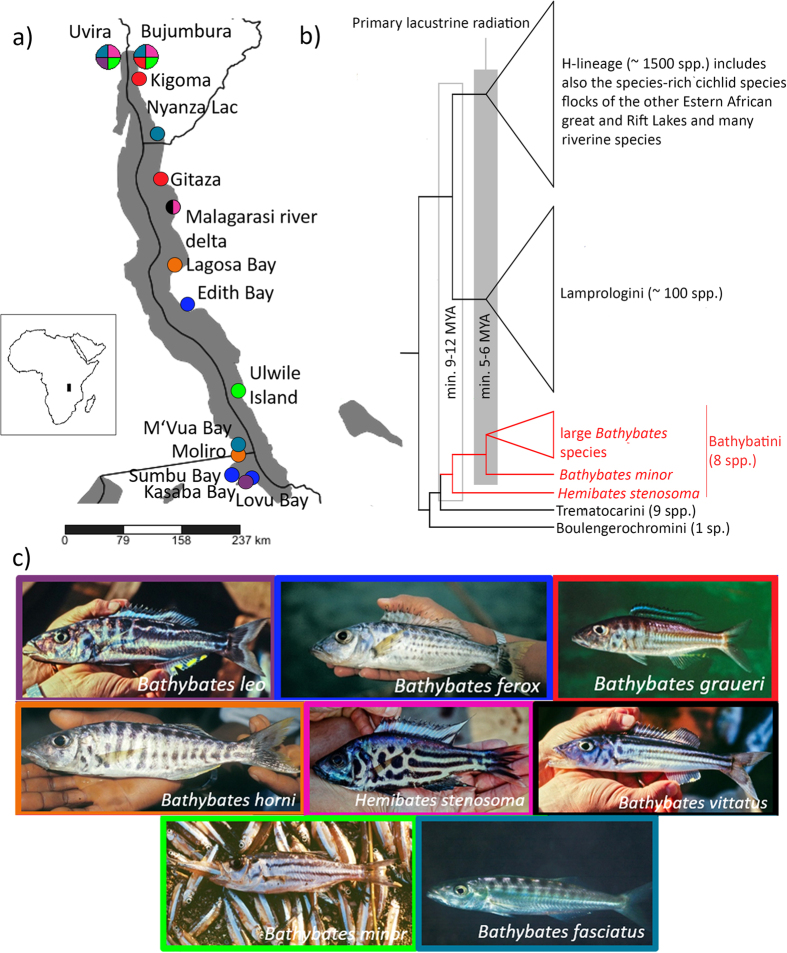
Host species information. (**a**) Geographical positions of sampling localities in Lake Tanganyika with indication of host species (pictures by Ad Konings). (**b**) Schematic phylogenetic tree of the Lake Tanganyika cichlid radiation, showing the phylogenetic position and relative divergence of the tribe Bathybatini and its major lineages^48^,^50^,^52^. (**c**) Host species pictures (Ad Konings). The map was created using SimpleMappr software v7.0.0. (available at http://www.simplemappr.net. Accessed February 20, 2016).

In total, 764 *Cichlidogyrus* specimens were retrieved and identified from 24 fish specimens belonging to six host species, namely *B. leo* Poll, 1956, *B. minor* Boulenger, 1906, *B. horni* Steindachner, 1911, *B. vittatus* Boulenger, 1914, *B. fasciatus* Boulenger, 1901 and *H. stenosoma* (Boulenger, 1901), making use of fresh material from an expedition in 2013 and of the historical ichthyological collections of the Royal Museum for Central Africa (Tervuren, Belgium) (Fig. 1; Table 2). All these *Cichlidogyrus* specimens were collected from the hosts’ gills. No monogeneans were found on *B. graueri* Steindachner, 1911 and *B. ferox* Boulenger, 1898. The overall prevalence on most of the species was 100%. The lowest prevalence was recorded on *B. leo* (25%). Infection intensity ranged from 1 to 263 individuals per gill chamber. This parameter was counted only for one gill chamber as one side needed to remain undamaged in museum specimens. Infection parameters are detailed in Table 2. They are only indicative because of the small sample size. All *Cichlidogyrus* specimens were identified as *C. casuarinus* based on the original description^15^ according to the corresponding shape and measurements of haptoral and male genital hardparts. The often slightly wider range of measurements is interpreted as a logical consequence of a larger sample size and the wider host and geographical range of the measured individuals (Table 3).

### Morphometric and geomorphometric assessment of intraspecific variation

**Figure 2:**
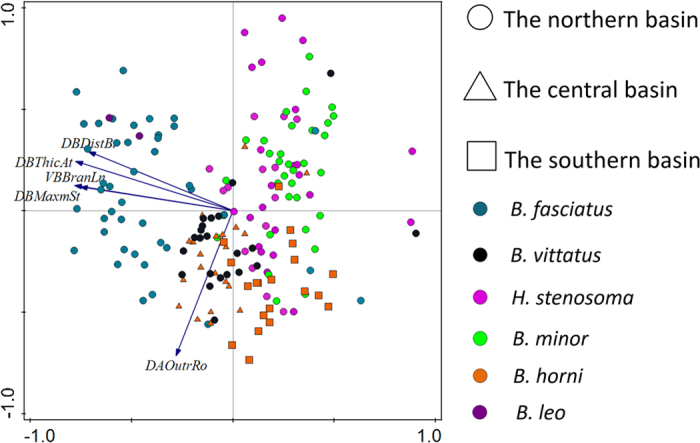
A biplot of PCA (first two axes) based on measurements of haptoral sclerotized structures only showing the five best fitting morphological characters selected by CANOCO. Symbols denote host species and their origin in each of the three subbasins of Lake Tanganyika. DALENGTO – Dorsal anchor, Length to notch, DATotlLn- Dorsal anchor, Total length, DBMaxmSt – Dorsal bar, Maximum straight width, VATotlLn – Ventral anchor, Total length, VBBranLn – Ventral bar, Branch length.

Principal component analysis (PCA) was performed on measurements taken on the haptoral hardparts of 182 individuals of *C. casuarinus* to assess intraspecific variation. The first PC explained 60% and the second 11% of the variation in our dataset. The shape of the bars had the highest contribution to PC1 whereas the size of one component of the dorsal anchor (outer root) was the main contributor to PC2. The resultant biplot graph, in which samples were grouped according to host species as well as to the three lake subbasins following Danley *et al*.^57^ (Table 2), showed some differentiation according to host species (Fig. 2). Individuals collected from *H. stenosoma* and *B. minor* clustered mostly along the positive side of the first axis. Specimens collected from *B. fasciatus* and *B. leo* had low values for this axis, with the exception of the three specimens coming from M’Vua Bay (southern basin). Another group was formed by parasites retrieved from *B. horni* and *B. vittatus*. These worms displayed lower values for the second axis. Most of the specimens coming from the central and the southern part of the lake displayed low values for the first axis while values for parasites collected in the northern part were widespread across the graph (Fig. 2). However, the fact that data from the central and southern parts of the lake are comprised only by specimens collected from *B. horni* could have influenced the result. Analyses of variance (ANOVA) provided information about intraspecific variation of *C. casuarinus* in copulatory tube and heel length (see Supplementary Tables S2 and 5). Box-plot graphs with the length of male copulatory organ structures are showed in Supplementary Figs S1 and S2. The analyses of copulatory tube length were based on 157 individuals while analyses of heel length were based on 149 individuals. Significant differences in copulatory tube length were observed between *C. casuarinus* from all of the host species except for *B. fasciatus* and *B. vittatus*. Comparisons of heel lengths showed a significant difference between *C. casuarinus* collected from *B. fasciatus* and the other host species, except for *B. vittatus*. There was no significant difference among individuals collected from *H. stenosoma, B. minor* and *B. horni.* No influence of geographical range was observed on the intraspecific variation in copulatory tube length. Individuals collected from the south of the lake differed significantly in heel length from specimens coming from the north.

**Figure 3:**
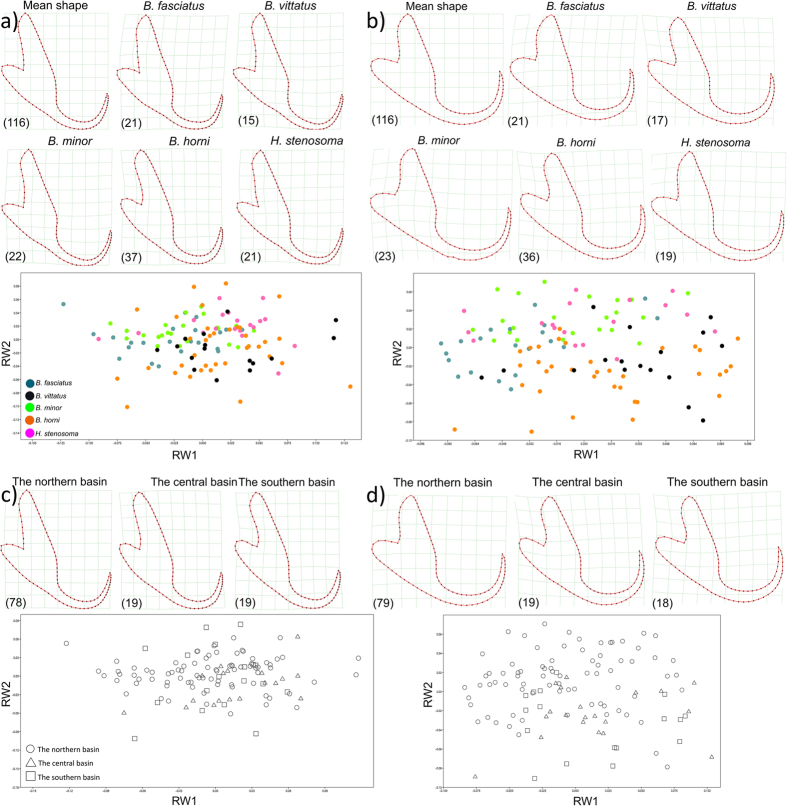
Scatterplots of the first two relative warps showing shape variation of the dorsal and ventral anchor with deformation grids (thin-plate) depicting mean anchor differences among groups. Symbols denote host species and sampling localities: (**a**) dorsal anchor, separation according to the host species; (**b**) ventral anchor, separation according to the host species; (**c**) dorsal anchor, separation according to the sampling localities; (**d**) ventral anchor, separation according to the sampling localities. The number of specimens investigated is indicated in brackets.

Intraspecific shape plasticity of *C. casuarinus* was analysed using landmarks and semilandmarks placed on one of the dorsal and ventral anchors. Specimens collected from *B. leo* were not included in the geomorphometric analysis because only two individuals were available. Scatterplots of relative warps showed some clustering according to host species, mainly along the second axis. Sample distribution along the first axis was caused by allometric effects, which were due to differences in the total size of the structures (individuals collected from *B. fasciatus* with the smallest ventral/dorsal anchor contrary to *C. casuarinus* from *B. horni* with the largest measured structure). Differences in the second axis were caused by variation in the shape of the anchors, for which no size effect could be found. For both analyses, specimens collected from *H. stenosoma, B. minor* and, to a lesser extent, *B. fasciatus* had relatively high values for the second axis. Values on the second axis were highly variable for *B. horni* and *B. vittatus* for the dorsal anchors, whereas they had low values on this axis for the ventral anchor. Based on the values of the bending energy (see Supplementary Table S6), individuals found on *H. stenosoma* seemed to have anchors that correspond most with the mean shape of both anchors in the dataset. The most divergent shape was displayed by specimens of *C. casuarinus* recorded from *B. vittatus* hosts (Fig. 3). The values for specimens collected in the northern part of Lake Tanganyika clustered mainly in the area with high values for the second relative warp. Specimens coming from southern and central localities tended to have lower values for this axis. This result was most evident in the shape of the ventral anchor but less so for the dorsal anchor (Fig. 3).

Based on the results of scatterplots, we defined two groups. The first was formed by the specimens collected from *H. stenosoma, B. minor* and *B. fasciatus.* The specimens recorded from *B. horni* and *B. vittatus* hosts were placed in a second group. Non-parametric Mann-Whitney U tests showed significant differences between these groups for both the dorsal (Z_1,117_ = −3.14122, P < 0.01) and the ventral anchor (Z = −3.59488, P < 0.001). Another test was performed to check for significant geographic differences in anchor shapes, comparing specimens collected in the north of the lake with those collected elsewhere. Whereas a significant difference was found between these groups in the shape of the ventral anchor (Z = −2.3227, P < 0.05), this was not the case for the shape of the dorsal anchor (Z = −1.77484, P > 0.05).

### Genetic species identification

All host specimens of which parasites were available for genetic analyses (*H. stenosoma, B. minor, B. fasciatus*) came from the very northern end of the lake. Genetic species identification of parasites was performed using three nuclear markers (28 S rDNA, 18 S rDNA, ITS-1) generally considered as suitable for monogenean species level determination^58^,^59^. The length of the successfully sequenced 28 S rDNA fragment ranged from 641 to 747 base pairs (bp). The 18 S rDNA fragment was 195–482 bp long, while the length of ITS-1 sequences ranged between 141 and 474 bp. In total, 27 sequences of 28 S rDNA and 25 sequences of 18 S + ITS1 rDNA were acquired. The *Cichlidogyrus* parasites shared an identical haplotype for all three rDNA regions and are hence confirmed to be conspecific. Formaldehyde fixation prevented the use of samples from the historical RMCA collections for the genetic part of this study.

### Population structure and past population size trajectories

**Figure 4:**
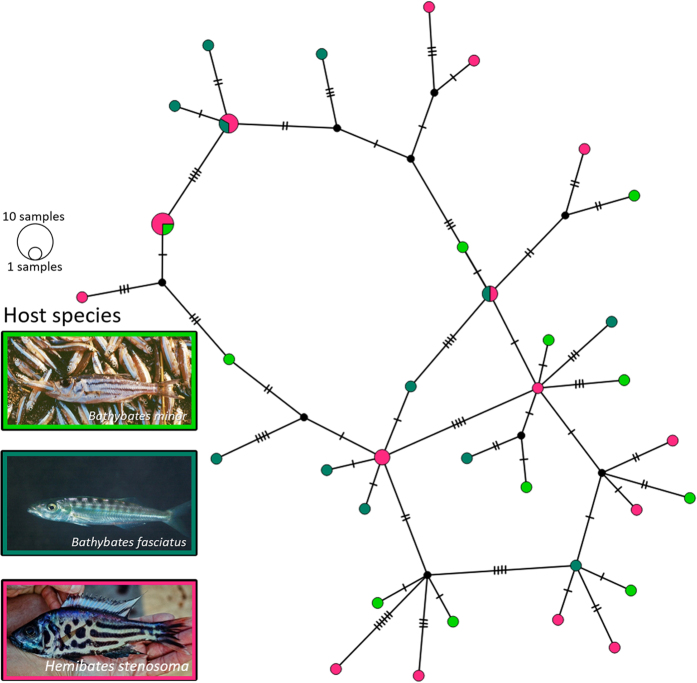
Haplotype network of *C. casuarinus* COI sequences (n = 42). The circles represent different haplotypes with size proportional to the number of individuals represented. Haplotypes are connected with lines, indicating number of mutations. Colours correspond with the host species (pictures by Ad Konings).

**Figure 5:**
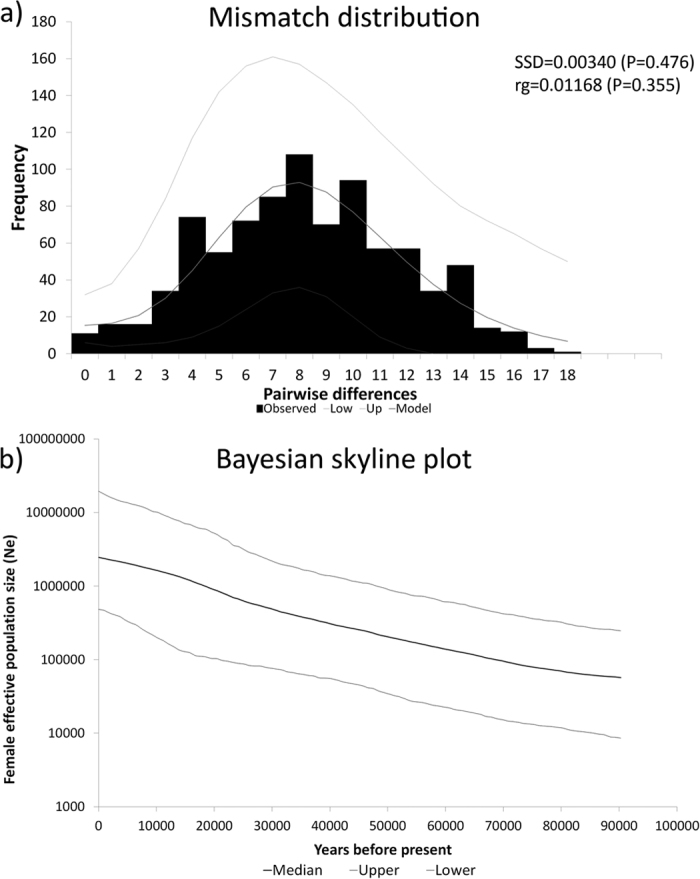
Demographic history of *Cichlidogyrus casuarinus.* (**a**) Mismatch distribution. The black bars show the observed frequency of pairwise differences. The grey lines refer to the expected distribution based on parameter estimates (plus 95% confidence limits) under a model of population growth. The sum of squared differences (SSD) and raggedness index (rg) and their respective P-values are given to describe the fit of the observed distribution to the expectations based on growth parameter estimates, as well as τ, the modal value of the mismatch distribution. (**b**) Bayesian skyline plot (BSP) based on 402 base pairs of COI sequences of *Cichlidogyrus casuarinus* showing the effective populations size through time, assuming a substitution rate of 10% per site per million years. The thick line represents the median values; the thin lines denote 95% highest posterior density (HPD) intervals.

Population structure was assessed using the mitochondrial marker COI. This region was used because of its fast rate of molecular evolution as compared to the nuclear sequences^60^. Since specimens suitable for DNA extraction were only available from the northern basin, a geographical effect could not be taken into account in the genetic analyses. The length of the COI sequences ranged from 466 to 1120 bp. Sequences for COI mtDNA were obtained from 42 individuals of *C. casuarinus* collected from three host species (*H. stenosoma, B. minor, B. fasciatus*), comprising 35 different haplotypes and containing 50 polymorphic sites. Analyses were based on a 402 bp fragment of COI. Haplotype and nucleotide diversity were estimated to be 0.987 and 0.02045, respectively. Genetic distance among haplotypes ranged from 0.2% to 4.7%. The haplotype network representing the relationships among *C. casuarinus* COI haplotypes is depicted in Fig. 4. There was no evident clustering according to host species and therefore no indication for cryptic diversity or incipient speciation. Moreover, a non-significant F_ST_ indicates no barriers between the groups defined by host species (see Supplementary Table S1), at least in northern Lake Tanganyika. The unimodal mismatch distribution with non-significant SSD (SSD = 0.00340, P = 0.476) and rg (rg = 0.01168, P = 0.355) indicated past population expansion of the *C. casuarinus* population (Fig. 5a). In addition, this result is well supported by the negative and significant value of Fu’s F_S_ (−24.01572, P < 0.001). Tajima’s D was negative, as expected for recent population growth, but not significantly different from zero (−1.03465, P = 0.142), which is probably due to the reduced power of Tajima’s D for detecting population expansion as compared to Fu’s F_S_^61^. Also the Bayesian Skyline Plot (BSP; Fig. 5b) indicated that *C. casuarinus* experienced population expansion in the recent past. The time to the most recent common ancestor (TMRCA) was dated to 144.4 KYA (95% HPD: 92.1–204.1 KYA). The onset of population expansion was dated to 87.0 KYA (95% CI: 51.4–167.0 KYA) based on the parameter τ = 6.994 (95% CI: 4.129–13.428) from the mismatch distribution (τ = 2 μt; where μ is the mutation rate per locus and t is the time since the onset of population growth).

## Discussion

The main aim of this study was to test for host-specificity of monogeneans in the non-littoral habitat of Lake Tanganyika. Genetic and morphological methods were used to answer questions about the diversity of the monogenean fauna occurring on deepwater fishes. Using multivariate statistical approaches, we investigated the host range and intraspecific variation of *Cichlidogyrus casuarinus*. Population-level analyses using mtDNA were performed to check whether host preference is driving speciation in *C. casuarinus*. This also allowed us to infer the species’ demographic history.

The previously proposed low host-specificity of *C. casuarinus* in the deepwater habitat^15^, which contrasts with the high host-specificity of many of its congeners in the littoral zone^8^, was supported by the fact that no variation was observed at the three rDNA regions in *C. casuarinus* sampled across different host species. Nuclear rDNA regions are considered to be suitable markers for species-level identification of monogeneans^59^,^62^. They have been used to show that allegedly generalist *Cichlidogyrus* species might actually comprise a complex of cryptic species, which are more host-specific than previously assumed^28^. However, our rDNA data confirm that *Cichlidogyrus* specimens infecting various bathybatine species are truly conspecific. Hence, we are dealing with an intermediate generalist species, parasitizing on a range of host-species within the same tribe^43^. Such weak host preference is probably an adaptation to the lower host availability in the deepwater realm, as has been suggested in previous studies on marine systems^4^,^5^,^6^. Therefore, *C. casuarinus* has evolved a different strategy compared to its congeners in the littoral zone (it is the only generalist species of *Cichlidogyrus* collected from the lake so far^15^), by broadening its host range, probably increasing the chance of contact and reducing that of extinction. The host range of *C. casuarinus* spans *Hemibates stenosoma* and the whole phylogenetic range of *Bathybates*. However, there are also species of *Bathybates* where monogenean infection has not been recorded yet (*B. graueri* and *B. ferox*). In Lake Tanganyika, 24 *Cichlidogyrus* species have already been described from 20 different host species^10^,^11^,^12^,^13^,^14^,^15^,^18^. Intermediate specialists were recorded in a previous study, namely *C. vandekerkhovei, C. makasai, C. centesimus* and *C. sturmbaueri* Vanhove, Volckaert & Pariselle, 2011 recorded from two to three *Ophthalmotilapia* species^13^ as well as *C. franswittei* Pariselle & Vanhove, 2015 infecting two species of *Pseudosimochromis*^14^. However, whereas past and/or ongoing hybridisation between *Ophthalmotilapia* species might explain their shared parasite species^65^, this does not seem to have been the case in the bathybatines. The divergence between them is ancient (Fig. 1b), and there is no evidence for any past or ongoing interspecific geneflow^49^,^53^. Hence, the lower host-specificity of *C. casuarinus* cannot be attributed to a shallow host phylogeny, confirming its more generalist lifestyle. Close morphological similarity of *C. casuarinus* with *C. nshomboi* and *C. centesimus*, which infect species from other Lake Tanganyika cichlid tribes (Boulengerochromini and Ectodini, respectively), was recorded^15^. These three monogenean species are the only known representatives of their genus exhibiting a spirally coiled thickening of the wall of the copulatory tube (see also Fannes *et al*.^66^). Together, they infect a variety of Tanganyika cichlids, with different feeding and reproductive strategies, occurring in different habitats and belonging to different tribes. This indicates that this morphotype of *Cichlidogyrus* is characterised by a rather broad niche.

Considering COI is the fastest evolving marker currently available for these monogeneans^59^, it was used to investigate the intraspecific variability of *C. casuarinus* in our study. Although the characterisation of *C. casuarinus* as an intermediate generalist species is well supported by ribosomal DNA, morphometric analyses of haptoral elements showed intraspecific variation, which was linked to host species. Based on the available knowledge about the member species of the Bathybatini, we could not discern a clear link between the morphological differentiation of *C. casuarinus* and host ecology (e.g., prey and habitat)^49^,^67^,^68^. Host body size and phylogenetic history could explain some of the groupings observed in the analyses of haptoral structures. Individuals collected from *H. stenosoma* and *B. minor*, which are the smallest species of the clade^68^ and which represent more basal lineages^49^,^53^, clustered together. Parasites originating from *B. horni, B. vittatus, B. leo* and *B. fasciatus* also clustered in our analysis. These host species stem from a phylogenetic lineage that also includes *B. ferox* and *B. graueri*^49^. Our data therefore suggest a correlation between morphological variation in *C. casuarinus* and the size and phylogenetic position of the host.

The most important morphometric features showing intraspecific variability in our dataset were maximum straight width, thickness at the middle and distance between auricles of the dorsal bar, branch length of the ventral bar and length of the outer root of the dorsal anchor. Although various sclerotized structures (heel length, length of copulatory tube, length of dorsal bar auricle) in other previously described *Cichlidogyrus* species exhibit a considerable size range^10^,^12^,^13^,^15^, the range observed in this study is wider. This difference could be explained by the increased geographical range and host range included in the present study compared to the original descriptions of parasites in Lake Tanganyika. Only one previous study^13^ also looked at some aspects on intraspecific morphometric variability of monogenean species in Lake Tanganyika, demonstrating intraspecific morphological variation of the MCO heel. The greatest infection intensity was observed on two relatively large cichlid species (*B. horni* and *B. vittatus*). This confirms the correlation between infection intensity and host body size^69^. However, individuals from another large host species included in our study, *B. fasciatus*, were not affected so severely by monogeneans. This discrepancy could be caused by limited sample size (random choice) or by massive infection in certain areas or times (the *B. horni* and *B. vittatus* specimens originated from 1949 and were collected at three different localities, see Table 2). In theory, prevalence and infection intensity could help us to identify the original and the more recent hosts of *C. casuarinus*^70^,^71^. Yet, it is hard to reliably quantify such parameters in view of the rarity of several of the host fishes^54^. Another way to establish which hosts have been colonized earlier would be through (co-)phylogenetic analyses. Although the observed groups based on parasite morphology correspond with the separate position of *H. stenosoma* and *B. minor* relative to the other species in the published host phylogeny^49^,^53^ unfortunately, our taxon coverage of Bathybatini was only exhaustive for morphological analysis, as museum specimens were unsuitable for molecular work. Moreover, the sequence data generated in this study did not consistently differ between parasites from different host species.

The geomorphometric approach suggested the existence of intraspecific shape variation in both dorsal and ventral anchors. Clustering along the relative warps axes shows almost the same sample distribution according to host species as the PCA of our set of linear haptoral measurements. Phenotypic changes of haptoral sclerites have already been described in many previous studies and are supposed to be influenced by a combination of host characteristics^72^,^73^ geographical origin^74^ and other environmental factors^77^,^78^. While some researchers prefer the haptoral region for reconstructing evolutionary history^28^,^58^, other investigations devote more attention to the reproductive organs^79^,^80^. We found significant intraspecific differences in certain parts of the male copulatory organ between parasites collected from different host species. Although this could be a sign of a possible reproductive barrier, it is known from this morphotype of Lake Tanganyika *Cichlidogyrus* that heel length can vary substantially within a species^13^. Moreover, geographic variation of reproductive and haptoral sclerotized structures was found by both morphometric techniques. However, unequal sampling of host species across different basins might have influenced our results. *Bathybates minor, B. fasciatus, B. leo, B. vittatus* and *H. stenosoma* were mostly or exclusively collected from the northern part of the lake, whereas the sample of hosts from the central and southern basins was dominated by *B. horni*.

The observed high haplotype diversity is consistent with a large population size of *C. casuarinus*^81^. Non-significant F_ST_ estimates suggested a lack of population genetic structure with respect to host species. Equally, there was no indication of ongoing speciation influenced by host preference apparent in the haplotype network. Its non-hierarchical topology indicated the absence of host related population structure. It has been suggested that a broad host range of morphometrically similar monogeneans can result from cryptic speciation processes^28^,^82^. However, in that case we should be able to recognise different haplotype variants corresponding with host preference: “choice matters”^55^. Since the intraspecific genetic variation was independent of host species, no cryptic or incipient speciation was evident in this system. Rather, the observed pattern of morphological variation seemed to be caused by phenotypic changes during ontogenetic development as an adaptation to the host or to the environment. Regarding the differences in MCO morphology, it is unclear how this may be influenced by the host. Poor correlation between genetic and morphometric variation may also be caused by limited sample size, only including COI sequences of *C. casuarinus* individuals from three host species or by a higher rate of genital morphological changes as compared to mutations in COI^55^. Even though there is no evidence for population structure according to host species or geographic origin based on COI fragments, future studies employing a large number of unlinked nuclear loci (i.e. generated by next-generation sequencing approaches) might reveal some population structure^85^,^86^. However, even if that was the case, it would indicate recent population splitting postdating the ancient divergence of the hosts, since the markers available for this study are generally used to distinguish closely related monogenean populations or species^8^,^55^.

Mismatch distribution, BSP and neutrality test all suggested past population expansion of *C. casuarinus*. These analyses were based on COI sequences and all indicated a recent increase in effective population size. However, only one of the neutrality tests (Fu’s F_S_) was significant. While Fu’s F_S_ compares expected and observed haplotype diversity and while it is sensitive to demographic expansion^87^, significantly negative values of Tajima’s D can result from positive selection, a bottleneck, or population expansion^88^. Compared to other neutrality test statistics, Tajima’s D has considerably lower power to detect population expansion^61^. Hence the data (non-significant negative D) were still compatible with population expansion. The reported lake level lowstand during the megadraught period ~100 KYA, when the water level dropped by up to 435 m below the present level (which was not enough to separate the lake into its three sub-basins^89^), reduced the inhabitable lake area considerably, even for pelagic and benthopelagic deepwater fish species. The subsequent lake level rise resulted in an expansion of the available habitat and might have triggered population expansion, a pattern reported for other pelagic and benthopelagic cichlid species from lakes Malawi and Tanganyika^90^,^91^. Moreover, recent work suggests congruent population expansion in some of the Bathybatini species (S. Koblmüller, unpublished data). Alternatively, recent host colonization or a bottleneck event might be responsible for the observed pattern^55^.

The present study is one of the most detailed investigations about intraspecific structure in monogeneans, and the first in an ancient lake. It confirmed the previously suggested decrease of host-specificity in *Cichlidogyrus* in the non-littoral habitat, with *C. casuarinus* as the first generalist species of the genus described from the lake. Therefore, it corroborates a pattern also observed in marine systems. There is a trophic relationship between bathybatine cichlids and economically much more important endemic clupeids of Lake Tanganyika’s open water. The predator-prey relationship was already suggested in previous studies to play an important role in host range expansion by transmission of parasites with a direct life cycle^92^. Therefore, it is recommended to also scrutinise these fisheries target species to assess the diversity and dynamics of parasites in the pelagic zone of this unique freshwater ecosystem. The lack of evidence for genetic population structure related to host preference in *C. casuarinus*, the significant intraspecific phenotypic plasticity influenced by the host and the reported population expansion of *C. casuarinus* suggest a high ability of (morphological) adaptation in this monogenean.

## Material and Methods

### Study area and sampling

Fish specimens (*Bathybates leo, B. minor, B. fasciatus, B. graueri* and *H. stenosoma*) were bought at several fish markets in the northern part of Lake Tanganyika, more specifically in the cities of Bujumbura and Uvira. Fishes were identified to the species level *in situ*. Gills were removed according to the standard protocol of Ergens and Lom^93^ and immediately preserved in pure ethanol. Some fresh gills were also inspected *in situ* for monogenean parasites under a stereomicroscope. Protocols were approved by the competent local authorities (mission statement 022/MINEURS/CRH-U/2013) and the Animal Care and Use Committee of Masaryk University, and carried out in accordance with permit CZ01308. To complete the taxon coverage and include geographical variation, fishes from the collection of the Royal Museum for Central Africa (Tervuren, Belgium) were also dissected (*B. ferox, B. horni, B. vittatus, B. fasciatus* and *H. stenosoma*) (Table 2, Fig. 1). Monogeneans that were to be used for morphometric analyses were mounted on a slide under a coverslip in Hoyer’s medium^94^. These specimens were deposited in the invertebrate collection of the RMCA (Table 2). Worms to be used for genetic work were mounted on a slide with a little drop of water under a cover slip after which pictures of the sclerotized structures were taken under phase contrast using an Olympus BX51 microscope and MicroImage analysis software 4.0. This procedure allowed for *post hoc* species-level identification of specimens of which the entire body was used for DNA extraction. Afterwards, these monogeneans were stored in 1.2 ml eppendorf tubes filled with 99.8% ethanol for subsequent DNA isolation.

### Morphometrics and geomorphometrics

Morphological characterization was based on the sclerotized structures of the parasite body; i.e. the opisthaptor and the genital parts. Measurements and photos were taken using an Olympus BX51 microscope with incorporated phase contrast at a magnification of 1000x (objective x100 immersion, ocular x10) with MicroImage 3.1. In total, 26 different features were measured on each individual. The terminology combined Řehulková *et al*.^38^ and Pariselle *et al*.^15^. To check for within-species variation in haptor morphology, a principal component analysis was performed on linear haptoral measurements of *C. casuarinus* monogenean parasites from different hosts and localities. For this, the length of pairs of hooks VI and VII was excluded because of the small sample size. This analysis was conducted in CANOCO 5.01^95^ on the basis of measurements of 21 selected morphological characters of the haptoral region. Missing data were replaced by the average value of each morphological character. ANOVA tests of MCO structures (only when data was available for more than 15 specimens collected per host species/locality) were performed in STATISTICA 12. To take possible geographical intraspecific variation into consideration, samples were also grouped into three basins according to Danley *et al*.^57^ (Table 2). The assumption of homogeneous variance within our sample groups was verified by Levene’s test. This prerequisite was only met in the case of the copulatory tube length and for groups defined by host species where Bonferroni’s post-hoc test was performed. Other analyses were therefore conducted using the non-parametric variant, namely Kruskal-Wallis ANOVA (see Supplementary Tables 2 and 5).

Geomorphometric analyses focuses on the visualisation of complex shape variation and provides an additional view to the classical morphometric study (linear measurements)^96^,^97^. Since a significant phylogenetic signal in anchor shape was detected in previous studies^98^, the ventral and dorsal anchors of *C. casuarinus* were digitalized using landmarks and semi-landmarks with TPSDIG2 software (Rohlf, 2006, TPS package, Stony Brook University). Positions and number of landmarks (5) and semi-landmarks (95) follow Vignon *et al*.^56^. Semi-landmarks were distributed in equal intervals. Generalized least square superpositions of landmark and semi-landmark coordinates was computed in TPSRELW (Rohlf, 2006, TPS package, Stony Brook University). The degree of shape deformation was quantified by estimating the minimal shape parameters (relative warps) needed to deform the consensus configuration to each specimen computed from partial warps^96^,^99^,^100^. As above, groups were defined in two ways: according to the host species and to the geographical origin of the specimen. To visualize mean shape anchor differences, thin-plate spline deformation grids were depicted in TPSSpline (Rohlf, 2006, TPS package, Stony Brook University). Parasites collected from *B. leo* were excluded from the analysis because of the small number of parasite specimens.

### Genetic species identification and intraspecific structure

To study the genetic diversity within *Cichlidogyrus casuarinus*, markers with varying rates of molecular evolution were used. These are: the 18 S ribosomal DNA (rDNA) gene, the 28 S rDNA gene, the first internal transcribed spacer region (ITS-1), and the mitochondrial cytochrome *c* oxidase subunit I gene (COI) (GenBank accession numbers: 28 S: KX007796-822, 18 S + ITS1: KX007775-95, COI: KX007823-64). Unfortunately, samples from the RMCA collections could not be used in the genetic part of this study because of fixation in formaldehyde. PCR conditions are mentioned in Supplementary Methods.

The analyses of population structure and demographic history within *C. casuarinus* were based on the COI sequences. COI was used because of its fast rate of molecular evolution as compared to the nuclear markers^59^,^60^. This allows for the detection of recent evolutionary events, such as possible incipient speciation as a result of host preference^55^. The number of haplotypes and polymorphic sites, haplotype diversity and nucleotide diversity^101^ were calculated using DnaSP 5.1^102^. Phylogenetic relationships among COI haplotypes were inferred by means of a Median Joining network^103^ in PopART 1.7^104^. Differentiation among pre-defined populations (according to host species) was estimated by F_ST_ in Arlequin 3.5.1.2^105^. To test for signals of past population expansion, a mismatch distribution and two different neutrality test statistics, Tajima’s D^88^ and Fu’s F_S_^106^ were calculated in Arlequin. Then the fit of the observed mismatch distribution to the expectations based on growth parameter estimates was evaluated by the sum of squared differences (SSD) and the raggedness index (rg). Significance was assessed with 10,000 permutations. Past population size trajectories were inferred employing a Bayesian coalescent approach (Bayesian skyline tree prior^107^) as implemented in BEAST 1.8.1^108^. We employed the model of evolution selected under the Bayesian information criterion in MEGA 6.06, assuming a strict molecular clock and a substitution rate of 10% per million years. Among monogeneans, a substitution rate estimate for COI is only available for *Gyrodactylus* (13.7–20.0% per million years;^109^). In view of the short generation time of *Gyrodactylus* compared to many other monogeneans^110^, it can be assumed that mutation rates of other monogeneans are somewhat lower^111^, and that the employed 10% per million years represents a reasonable approximation^8^. Two independent MCMC runs of 10 million generations each at a sampling frequency of 1,000 were conducted, with a burn-in of the first 10% of sampled generations. The number of grouped intervals was set to 5. Verification of effective sample sizes (ESS > 200 for all parameters), trace of MCMC runs and visualisation of past population size changes were done in Tracer 1.6 (Rambaut A, Suchard MA, Drummond AJ. 2014. Tracer v1.6, available from http://tree.bio.ed.ac.uk/software/tracer/).

## Additional Information

**Accession Codes**: The sequences obtained were deposited in NCBI GenBank under accession numbers KX007775-864.

**How to cite this article**: Kmentová, N. *et al*. Reduced host-specificity in a parasite infecting non-littoral Lake Tanganyika cichlids evidenced by intraspecific morphological and genetic diversity. *Sci. Rep.* **6**, 39605; doi: 10.1038/srep39605 (2016).

**Publisher's note:** Springer Nature remains neutral with regard to jurisdictional claims in published maps and institutional affiliations.

## Supplementary Material

Supplementary Information

## Figures and Tables

**Table 1:** List of the 24 monogenean species of *Cichlidogyrus* reported in Lake Tanganyika with host specification^10^,^12^,^14^,^15^,^42^,^112^,^113^.

Monogenean species	Host species	Host-specificity^43^
*Cichlidogyrus. attenboroughi* Kmentová, Gelnar, Koblmüller & Vanhove, 2016	*Benthochromis horii* Poll, 1948	strict specialist
*C. banyankimbonai* Pariselle & Vanhove, 2015	*Simochromis diagramma* (Günther, 1894)	strict specialist
*C. brunnensis* Kmentová, Gelnar, Koblmüller & Vanhove, 2016	*Trematocara unimaculatum* Boulenger, 1901	strict specialist
*C. buescheri* Pariselle & Vanhove, 2015	*Interochromis loocki* (Poll, 1949)	strict specialist
*C. casuarinus* Pariselle, Muterezi Bukinga & Vanhove, 2015	*Bathybates minor* Boulenger, 1906; *B. fasciatus* Boulenger, 1901; *B. vittatus* Boulenger, 1914 Potentially also on *B. leo* Poll, 1956 and *Hemibates stenosoma* (Boulenger, 1901)	**intermediate generalist?**
*C. centesimus* Vanhove, Volckaert & Pariselle, 2011	*Ophthalmotilapia ventralis* (Boulenger, 1898); *O. nasuta* (Poll & Matthes, 1962); *O. boops* (Boulenger, 1901)	intermediate specialist
*C. frankwillemsi* Pariselle & Vanhove, 2015	*Pseudosimochromis curvifrons* (Poll, 1942)	strict specialist
*C. franswittei* Pariselle & Vanhove, 2015	*P. marginatus* (Poll, 1956); *P. curvifrons*	intermediate specialist
*C. georgesmertensi* Pariselle & Vanhove, 2015	*P. babaulti* (Pellegrin, 1927)	strict specialist
*C. gillardinae* Muterezi Bukinga, Vanhove, Van Steenberge & Pariselle, 2012	*Astatotilapia burtoni* (Günther, 1894)	strict specialist
*C. gistelincki* Gillardin, Vanhove, Pariselle, Huyse & Volckaert, 2012	*Ctenochromis horei* (Günther, 1894)	strict specialist
*C. irenae* Gillardin, Vanhove, Pariselle, Huyse & Volckaert, 2012	*Gnathochromis pfefferi* (Boulenger, 1898)	strict specialist
*C. makasai* Vanhove, Volckaert & Pariselle, 2011	*Opthalmotilapia ventralis* (Boulenger, 1898); *O. nasuta* (Poll Matthes, 1932); *O. boops* (Boulenger, 1901)	intermediate specialist
*C. mbirizei* Muterezi Bukinga, Vanhove, Van Steenberge & Pariselle, 2012	*Oreochromis tanganicae* (Günther, 1894), *O. niloticus, O. mossambicus*	intermediate specialist
*C. mulimbwai* Muterezi Bukinga, Vanhove, Van Steenberge & Pariselle, 2012	*Tylochromis polylepis* (Boulenger, 1900)	strict speciliast
*C. muterezii* Pariselle & Vanhove, 2015	*S. diagramma*	strict specialist
*C. muzumanii* Muterezi Bukinga, Vanhove, Van Steenberge & Pariselle, 2012	*T. polylepis*	strict specialist
*C. nshomboi* Muterezi Bukinga, Vanhove, Van Steenberge & Pariselle, 2012	*Boulengerochromis microlepis* (Boulenger, 1899)	strict specialist
*C. raeymaekersi* Pariselle & Vanhove, 2015	*S. diagramma*	strict specialist
*C. schreyenbrichardorum* Pariselle & Vanhove, 2015	*I. loocki*	strict specialist
*C. steenbergei* Gillardin, Vanhove, Pariselle, Huyse & Volckaert, 2012	*Limnotilapia dardennii* (Boulenger, 1899)	strict specialist
*C. sturmbaueri* Vanhove, Volckaert & Pariselle, 2011	*O. ventralis; O. nasuta*	intermediate specialist
*C. vandekerkhovei* Vanhove, Volckaert & Pariselle, 2011	*O. ventralis*; *O. nasuta;* *O. boops*	intermediate specialist
*C. vealli* Pariselle & Vanhove, 2015	*I. loocki*	strict specialist

Terminology: Strict specialist – infecting only a single host species, intermediate specialist – infecting two or more congeneric host species, intermediate generalist – infecting non-congeneric host species from the same tribe, generalist – infecting two or more hosts from different tribes.

**Table 2:** An overview of host spe cies examined for *Cichlidogyrus* parasites with localities and infection parameters.

Host species (host maximum size^68^, cm)	Locality (geographic coordinates)	Locality – basins^57^ (date of sampling)	Number of fish specimens (accession number in RMCA)	Number of *Cichlidogyrus* individuals (accession numbers in RMCA)	Prevalence (%)	Infection intensity/one gill chamber	Abundance (range)
*Bathybates fasciatus* (39, 7)	Uvira (3°22′S 29°08′E)	The northern basin (9/9/2013)	1 (MRAC 2016-22-P)	12 (MRAC 37926-8)	100	6	6
	Bujumbura (3°23′S 29°22′E)	The northern basin (4/9/2013)	3 (MRAC 2016-22-P)	42 (MRAC 37921-5)	100	7.7	7.7 (1–19)
	M’Vua Bay (08°05′S-30°34′E)	The southern basin (23/3/1947)	2 (MRAC 112235-242 A, 115)	3 (MRAC 37898-9)	50	1.5	1.5 (0–3)
	Nyanza Lac (04°20′S-29°35′E)	The northern basin (1/1/1937)	3 (MRAC 54746-60 A, B, C)	7 (MRAC 37900-3)	66.6	3.5	2.3 (0–4)
*Bathybates horni* (27, 2)	Moliro (08°13′S-30°35′E)	The southern basin (12/3/1947)	1 (MRAC 112481)	263 (MRAC 37847, 49-73)	10	263	263
	Lagosa Bay (05°57′S-29°51′E)	The central basin (11/4/1947)	1 (MRAC 112484)	162 (MRAC 37827-46, 48)	100	162	162
*Bathybates leo* (26, 0)	Uvira	The northern basin (9/9/2013)	4 (−)	2 (MRAC 37758-9)	25	1	162
	Kasaba Bay (-08°31′S-30°39′E)	The southern basin (23/11/1995)	2 (MRAC 99-31P-896-904)	0	0	0	0
	near Malagarasi River delta (05°14′S-29°45′E and 05°13′S-29°43′E)	The northern basin (26/2/1947)	1 (112492-496)	0	0	0	0
*Bathybates minor* (20, 5)	Bujumbura	The northern basin (4/9/2013)	7 (MRAC 2016-22-P)	50 (MRAC 37904-8)	71.4	5	3.55 (0–9.5)
	Ulwile Island (07°25′ S-30°34′ E)	The central basin (4/9/2013)	1 (2016-22-P)	8 (MRAC 37909-14)	100	4	4
*Bathybates ferox* (38, 5)	Sumbu Bay (08°31′S-30°29′E)	The southern basin (31/3/1947)	3 (MRAC 112187-97 A, B, F)	0	0	0	0
	Lovu Bay (08°34′S-30°44′E)	The southern basin (26/3/1947)	1 (MRAC 112175-80)	0	0	0	0
	Edith Bay (06°30′S-29°55′E)	The central basin (14/2/1947)	3 (MRAC 112152-62 A, B, C)	0	0	0	0
*Hemibates stenosoma* (26, 0)	Bujumbura	The northern basin (25/9/2013)	4 (MRAC 2016-22-P)	28 (MRAC 37915-6)	75	4.7	3.5 (0–8)
	Uvira	The northern basin (9/9/2013)	4 (MRAC 2016-22-P)	36 (MRAC 37917-20)	100	4.5	4.5 (2.5–7.5)
	near Malagarasi River delta	The northern basin (22/5/1947)	1 (MRAC 112136)	27 (MRAC 37891-7)	100	27	27
*Bathybates viitatus* (42, 0)	near Malagarasi River delta	The northern basin (26/2/1947)	1 (MRAC 112489)	124 (MRAC 37874-90)	100	124	124
*Bathybates graueri* (30, 0)	Bujumbura	The northern basin (25/9/2013)	7 (MRAC 2016-22-P)	0	0	0	0
	Uvira	The northern basin (9/9/2013)	6 (MRAC 2016-22-P)	0	0	0	0
	Kigoma (04°52′S- 29°38′E)	The northern basin (10/1/1947)	3 (MRAC 112430-452)	0	0	0	0
	Gitaza (03°37′ S-29°20′E)	The northern basin (20/10/1995)	1 (MRAC 95-98-P-253-62)	0	0	0	0

**Table 3:** Comparison of measurements performed on *C. casuarinus* haptoral and genital hardparts between the present study and the original description^15^ (a – mean value ± standard deviation, b – range).

Parameters (μm)	*C. casuarinus* (n = 182)	*C. casuarinus* Pariselle *et al*.^15^ (n = 35)
Total length	628.7 ± 93.2^a^ (n = 83); (379.1–1003.4)^b^	915 (n = 19); (766–1105)
Ventral anchor		
Total length	51 ± 4.7 (n = 158); (38.3–62.5)	51 ± 2.5 (n = 35); (47–59)
Length to notch	41.7 ± 3.8 (n = 157); (32.1–49.9)	43 ± 1.7 (n = 35); (39–47)
Inner root length	16.4 ± 2.6 (n = 156); (10.1–21.6)	17 ± 1.6 (n = 35); (12–19)
Outer root length	9.5 ± 2 (n = 154); (5.1–17.3)	8 ± 1.5 (n = 35); (5–11)
Point length	16.3 ± 2.1 (n = 157); (10.9–22.8)	16 ± 1.5 (n = 35); (12–20)
Dorsal anchor		
Total length	56.7 ± 5.5 (n = 156); (40–73.8)	58 ± 2.8 (n = 32); (52–64)
Length to notch	39.6 ± 3.6 (n = 156); (29.7–46.8)	40 ± 2.0 (n = 32); (35–44)
Inner root length	22.1 ± 3.1 (n = 156); (15.5–31.3)	24 ± 1.9 (n = 32); (20–27)
Outer root length	8.4 ± 2 (n = 154); (2.5–14.1)	8 ± 1.3 (n = 32); (6–11)
Point length	14.2 ± 1.6 (n = 154); (8.9–18.2)	15 ± 0.8 (n = 32); (13–17)
Ventral bar		
Branch length	64.2 ± 8.3 (n = 157); (43.4–90.3)	59 ± 3.2 (n = 39); (54–67)
Branch maximum width	9.5 ± 1.8 (n = 164); (5.1–14.5)	9 (n = 20); (7–12)
Dorsal bar		
Maximum straight width	79.6 ± 13.1 (n = 138); (54.7–115.2)	71 (n = 20); (64–85)
Thickness at midlength	15.4 ± 3.7 (n = 174); (9.3–39.2)	15 (n = 15); (12–20)
Distance between auricles	33.7 ± 7 (n = 169); (21.8–55.4)	30 (n = 20); (23–40)
Auricle length	18.7 ± 3.2 (n = 154); (7.8–28.6)	17 ± 1.8 (n = 40); (13–23)
Hooks		
Pair I	33.8 ± 4.3 (n = 157); (22–49.4)	30 ± 1.2 (n = 30); (27–33)
Pair II	22.3 ± 2.6 (n = 129); (11.8–33.5)	—
Pair III	23.7 ± 2.8 (n = 121); (10.6–29.9)	—
Pair IV	25.8 ± 2.7 (n = 111); (13.8–31.2)	—
Pair V	10.8 ± 0.9 (n = 109); (6.2–13.8)	11 (n = 17); (10–12)
Pair VI	26.3 ± 3.8 (n = 69); (15.9–33.1)	—
Pair VII	27.4 ± 3.6 (n = 64); (14.2–32.5)	—
Pair II, III, IV, VI, VII average size	24.6 ± 3.5 (n = 494); (10.6–33.5)	23 ± 1.9 (n = 120); (19–28)
Copulatory tube straight length	37 ± 3.1 (n = 163); (29.7–43.2)	37 (n = 20); (34–44)
Accessory piece curved length	32.8 ± 5.8 (n = 27); (33.1–103.2)	31 (n = 20); (26–38)
Heel straight length	60 ± 15.7 (n = 157); (25.7–46.9)	47 (n = 20); (40–59)
Vagina curved length	56.3 ± 12.7 (n = 34); (38.1–83.1)	46 (36–59)
Vagina maximum width	12 ± 2.2 (n = 49); (7.7–16.5)	7 (5–8)
