# Supplementary material for: Reduced host-specificity in a parasite infecting non-littoral Lake Tanganyika cichlids evidenced by intraspecific morphological and genetic diversity
Source: Sci Rep. 2016 Dec 22;6:39605. doi: 10.1038/srep39605 (PMC5177900; doi:10.1038/srep39605)
Supplement: Supplementary Information [file srep39605-s1.pdf]

Reduced host-specificity in a parasite infecting non-littoral Lake Tanganyika cichlids evidenced by intraspecific morphological and genetic diversity

Supplementary figures

Nikol Kmentová, Milan Gelnar, Monika Mendlová, Maarten Van Steenberge, Stephan Koblmüller, Maarten P. M. Vanhove

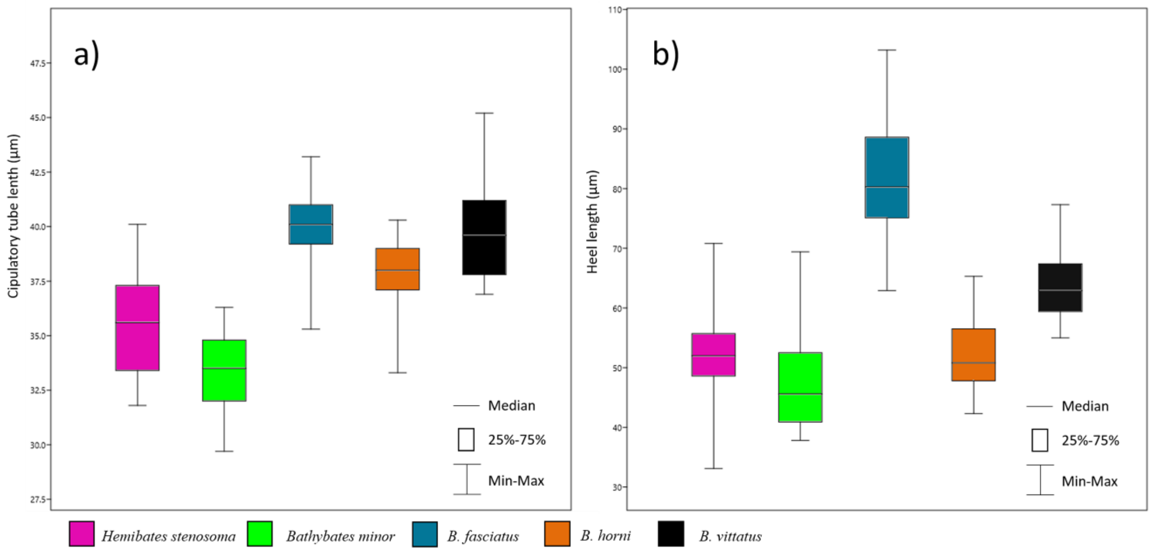

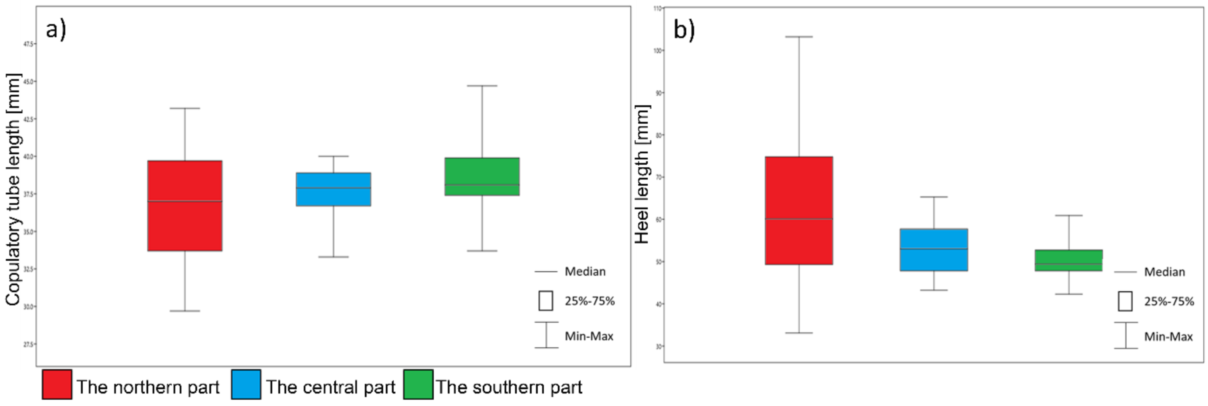

Figure captions

Supplementary Figure S1: Box-plot graph with male copulatory organ structures defined by host species: a) copulatory tube length; b) heel length.

Supplementary Figure S2: Box-plot graph with male copulatory organ structures defined by localities: a) copulatory tube length; b) heel length.

Supplementary tables

Supplementary Table S1: Pairwise F_{ST} estimates among pre-defined subpopulations.

**Subpopulation**		**F_{ST} (p-value)**
B. minor	*B. fasciatus*	-0.02477 (P>0.05)
B. minor	*H. stenosoma*	-0.01011 (P>0.05)
B. fasciatus	*H. stenosoma*	-0.03316 (P>0.05)

Supplementary Table S2: Bonferroni‘s test results of *C. casuarinus* copulatory tube length among groups defined by host species.

	**Bonferroni's post-hoc test, variable: copulatory tube length ($F_{4,155}$=74.2908, P<0.005) p-values**				
**Host species**	1	2	3	4	5
1 (n=34)		0.000131	0	0.000003	0
2 (n=35)	0.000131		0	0	0
3 (n=36)	0	0		0.000026	1
4 (n=35)	0.000003	0	0.000026		0.004808
5 (n=17)	0	0	1	0.004808	

1- *H. stenosoma* 2 – *B. minor*, 3 – *B. fasciatus*, 4 – *B. horni*, 5 – *B. vittatus*, n=number of specimens.

Supplementary Table S3: Kruskal-Wallis test results of *C. casuarinus* heel length among groups defined by host species.

	**Kruskal-Wallis test, variable: heel length (KW-$H_{4,151}$=100.7837, P<0.005) p-values**				
**Host species**	1	2	3	4	5
1 (n=29)		0.582163	0	1	0.011613
2 (n=33)	0.582163		0	1	0.000005
3 (n=36)	0	0		0	0.102135
4 (n=34)	1	1	0		0.002681
5 (n=20)	0.011613	0.000005	0.102135	0.002681	

1- *H. stenosoma* 2 – *B. minor*, 3 – *B. fasciatus*, 4 – *B. horni*, 5 – *B. vittatus*, n=number of specimens.

Supplementary Table S4: Kruskal-Wallis test results of *C. casuarinus* copulatory tube length among groups defined by sampling locality.

	**Kruskal-Wallis test, variable: copulatory tube length (KW-$H_{2,165}$=3.6308, P>0.05)** **p-values**		
**Locality**	1	2	3
1 (n=124)		0.641272	0.094859
2 (n=20)	0.641272		1
3 (n=20)	0.094859	1	

1 - The northern basin, 2- The central basin, 3 - The southern basin, n=number of specimens.

Supplementary Table S5: Kruskal-Wallis test results of *C. casuarinus* heel length among groups defined by sampling locality.

	**Kruskal-Wallis test, variable: heel length (KW-$H_{2,164}$=14.7814, P<0.005)** **p-values**		
**Locality**	1	2	3
1 (n=122		0.120183	0.001343
2 (n=20)	0.120183		0.855954
3 (n=20)	0.001343	0.855954	

1 - The northern basin, 2- The central basin, 3 - The southern basin, n=number of specimens.

Supplementary Table S6: Bending energy required to deform the reference shape of dorsal and ventral anchor, respectively, to the particular shape. Groups are defined by the host species of *C. casuarinus.*

		**Bending energy**			
**Host species**	Dorsal anchor	Ventral anchor	Localities	Dorsal anchor	Ventral anchor
H. stenosoma	0.02940	0.03103	northern basin	0.00476	0.00504
B. fasciatus	0.03270	0.03250	central basin	0.02420	0.03542
B. horni	0.02423	0.02534	southern basin	0.04093	0.03360
B. minor	0.03322	0.04955			
B. vittatus	0.03686	0.02157			

Supplementary methods

DNA extraction, PCR conditions and alignment

DNA extraction was conducted according to Kmentová (2015)[1]. Part of the 28S region of the ribosomal DNA was amplified using primers C1 (5'-ACCCGCTGAATTTAAGCAT-3') and

D2 (5’-TGGTCCGTGTTTCAAGAC-3’)[2] in a Mastercycler Thermocycler. Each amplification reaction contained two units of *Taq* Polymerase, 1X buffer containing 0.1 mg/ml Bovine Serum Albumine (BSA), 1.5 mM $MgCl_2$, 0.2 mM dNTPs, 0.5 µM of each primer and 30 ng of genomic DNA in a total reaction volume of 30 µl. The PCR protocol started with initial denaturation at 94°C for 2 min followed by 39 cycles of 20 sec at 94°C, 30 sec at 58°C and 1 min 30 sec at 72°C and ended with a final elongation for 10 min at 72°C. The primers S1 (5’-ATTCCGATAACGAACGAGACT-3’)[3] and IR8 (5’-GCAGCTGCGTTCTTCATCGA-3’)[4] were used to amplify part of the 18S gene and the entire ITS-1 region. Each amplification reaction contained 1.5 unit of *Taq* Polymerase, 1X buffer containing 0.1 mg/ml BSA, 1.5 mM $MgCl_2$, 200 mM dNTPs, 0.5 mM of each primer and 30 ng of genomic DNA in a total reaction volume of 30 µl under the following conditions: 2 min at 94°C, 39 cycles of 1 min at 94°C, 1 min at 53°C and 1 min and 30sec at 72°C, and finally 10 min at 72°C. Part of the COI gene was amplified using ASmit1 (5’-TTTTTTGGGCATCCTGAGGTTTAT-3’)[5] combined with Schisto3 (5’-TAATGCATMGGAAAAAAACA-3’)[6], and with ASmit2 (5’-TAAAGAAAGAACATAATGAAAATG-3’)[5] in case of nested PCR. The first amplification reaction with ASmit1 and Schisto3 primers contained 24 µl of PCR mix (one unit of *Taq* Polymerase, 1X buffer containing 2 mM $MgCl_2$, 0.2 mM dNTPs, 0.8 mM of each primer) with 30 ng of genomic DNA in a total reaction volume of 25 µl and was performed under the following conditions: initial denaturation at 95°C for 5 min and then 40 cycles of 1 min at 94°C, 1 min at 50°C and 1 min at 72°C, and final elongation for 7 min at 72°C. The nested PCR with ASmit1 and ASmit2 primers followed the same protocol as the first one. To increase amplification success another set of COI primers was used. The first PCR reaction was performed with the primer combination Mono5 (5'-TAATWGGTGGKTTTGGTAA-3') and Mono3 (5'-TAATGCATMGGAAAAAAACA-3')[7] and contained 23 µl of 1.5 unit of *Taq*

Polymerase, 1X buffer containing 0.1 mg/ml BSA, 1.5 mM $MgCl_2$, 0.2 mM dNTPs and 0.5 mM of each primer and 2 µl of 30 ng of genomic DNA in a total reaction volume of 25 µl. Amplification was performed carrying out the following PCR program: 5 min at 95°C followed by 40 cycles of 30 sec at 94°C, 30 sec at 44°C, 2 min at 72°C, and finally 7 min at 72°C. A nested PCR reaction was carried out with primers Mono5 and Mono3-int (5'-ACATAATGAAARTGAGC-3')[7] using the same protocol as in the first amplification. PCR products were purified using the High Pure PCR Product Purification Kit (Roche) according to the manufacturer's instructions. Identical primers as in amplification reactions were used for sequencing together with a Big Dye Chemistry Cycle Sequencing Kit 3.1. Obtained nucleic acid sequences were aligned using MUSCLE 3.2[8] under default distance measures and sequence weighting schemes, implemented in MEGA 6.06[9]. Sequences and their alignment were visually inspected and corrected using the same software.

References

1. Kmentová, N. Monogenean parasites from deepwater Lake Tanganyika cichlids: reduced specificity or hidden diversity? (MSc thesis, Masaryk University, 2015).

2. Hassouna, N., Michot, B., Bachellerie, J. & Narbonne, D. The complete nucleotide sequence of mouse 28S rRNA gene. Implications for the process of size increase of the large subunit rRNA in higher eukaryotes. *Nucleic Acids Res.* **12,** 3563–3583 (1984).

3. Matějusová, I., Gelnar, M., McBeath, A. J. A., Collins, C. M. & Cunningham, C. O. Molecular markers for gyrodactylids (Gyrodactylidae: Monogenea) from five fish families (Teleostei). *Int. J. Parasitol.* **31,** 738–745 (2001).

4. Šimková, A., Plaisance, L., Matějusová, I., Morand, S. & Verneau, O. Phylogenetic relationships of the Dactylogyridae Bychowsky, 1933 (Monogenea: Dactylogyridea): The need for the systematic revision of the Ancyrocephalinae Bychowsky, 1937. *Syst.*

Parasitol. **54,** 1–11 (2003).

5. Littlewood, D. T. J., Rohde, K. & Clough, K. a. Parasite speciation within or between host species? - Phylogenetic evidence from site-specific polystome monogeneans. *Int. J. Parasitol.* **27,** 1289–1297 (1997).

6. Lockyer, A. E. *et al.* The phylogeny of the Schistosomatidae based on three genes with emphasis on the interrelationships of *Schistosoma* Weinland, 1858. *Parasitology* **126,** 203–24 (2003).

7. Plaisance, L., Rousset, V., Morand, S. & Littlewood, D. T. J. Colonization of Pacific islands by parasites of low dispersal ability: Phylogeography of two monogenean species parasitizing butterflyfishes in the South Pacific Ocean. *J. Biogeogr.* **35,** 76–87 (2008).

8. Edgar, R. C. MUSCLE: Multiple sequence alignment with high accuracy and high throughput. *Nucleic Acids Res.* **32,** 1792–7 (2004).

9. Tamura, K., Stecher, G., Peterson, D., Filipski, A. & Kumar, S. MEGA6: Molecular Evolutionary Genetics Analysis version 6.0. *Mol. Biol. Evol.* **30,** 2725–2729 (2013).
